# Hepatocellular Carcinoma: A Critical Complication in Patients Treated with Pyridoxal Phosphate

**DOI:** 10.1055/a-2773-6076

**Published:** 2026-01-06

**Authors:** Marion M. Brands, Chloé de Puyraimond, Sidney M. Gospe Jr, Manuel M. Schiff, Bregje Jaeger, Bart G. Koot, Martine F. Raphael, Charlotte M. Lubout, Bertrand Soto, Julian Delanne, Apolline Imbard, John Zempel, Kathelijne C. Kraal, René Scheenstra, Peter T. Clayton

**Affiliations:** 1 Department of Pediatrics, Division of Metabolic Diseases, Emma Children's Hospital, Amsterdam Gastroenterology Endocrinology Metabolism, Amsterdam UMC location University of Amsterdam, Amsterdam, The Netherlands; 2 Department of Pediatrics, Reference Center for Inborn Errors of Metabolism, Necker Hospital, APHP, Université Paris-Cité, G2m Network, MetabERN, Paris, France; 3 Departments of Neurology and Pediatrics, University of Washington, Seattle, Washington, United States; 4 Department of Pediatrics, Duke University, Durham, North Carolina, United States; 5 INSERM UMR _S1163, Institut Imagine, Université Paris-Cité, Paris, France; 6 Department of Child Neurology, Emma's Children's Hospital, Amsterdam UMC, Amsterdam, The Netherlands; 7 Department of Pediatric Gastroenterology and Nutrition, Emma Children's Hospital, Amsterdam University Medical Centers, Location Academic Medical Center, University of Amsterdam, Netherlands; 8 Department of Pediatric Oncology, Emma Children's Hospital, Amsterdam UMC Location University of Amsterdam, Amsterdam, The Netherlands; 9 Department of Metabolic Diseases, Beatrix Children's Hospital, University Medical Center Groningen, University of Groningen, Groningen, the Netherlands; 10 Department of Pediatrics, CH Auxerre, Auxerre, France; 11 Department of Genetics, CHU Dijon Bourgogne, Dijon, France; 12 Department of Biochemistry, Hôpital Necker-Enfants Malades, APHP, Paris, France; 13 Département Médicaments et Technologies pour La Santé (DMTS), Paris-Saclay University, CEA, Gif-sur-Yvette, France; 14 Departments of Neurology and Pediatrics, Washington University, St. Louis, Missouri, United States; 15 Princess Máxima Center for Pediatric Oncology, Utrecht, The Netherlands; 16 Division of Pediatric Gastroenterology and Hepatology, Department of Pediatrics, University Medical Center Groningen, University of Groningen, Groningen, The Netherlands; 17 Inborn Errors of Metabolism, Genetics and Genomic Medicine, UCL Great Ormond Street Institute of Child Health, London, United Kingdom

**Keywords:** B6 epilepsy, liver toxicity, risk for malignancy, Epilepsy, Neurometabolics

## Abstract

**Background:**

Pyridoxal-5′-phosphate (PLP) is in most patients the effective treatment for pyridox(am)ine-5′-phosphate oxidase (PNPO) deficiency, a rare autosomal recessive cause of neonatal-onset developmental and epileptic encephalopathy. Although generally considered safe, long-term high-dose PLP exposure may have hepatotoxic effects, particularly in the absence of pharmaceutical-grade formulations.

**Methods:**

We report a series of four pediatric patients with vitamin B6–dependent epilepsy who received long-term PLP therapy. Two had genetically confirmed PNPO deficiency, and two were later diagnosed with ALDH7A1 deficiency. All received high-dose oral PLP, with frequent changes in formulation due to availability issues.

**Results:**

Three of the four patients developed hepatocellular carcinoma after several years of PLP treatment; one developed fully reversible severe hepatotoxicity. The shared exposure to prolonged high-dose PLP across all affected patients, despite differing metabolic conditions, suggests a possible role for PLP toxicity independent of the underlying metabolic disorder. Known toxic mechanisms include mitochondrial dysfunction, Schiff base–mediated protein modification, and accumulation of reactive PLP degradation products. In two patients, the total PLP dose was successfully reduced by over 30% through increasing administration frequency, without loss of seizure control.

**Conclusion:**

These findings raise significant concerns about the long-term hepatic safety of oral PLP in patients with vitamin B6–dependent epilepsies. As intravenous PLP is unfeasable for lifelong therapy, there is an urgent need for standardized, high-quality PLP preparations and exploration of alternative delivery routes such as intranasal administration. Regular hepatic monitoring should be implemented in all patients receiving chronic PLP therapy.

## Introduction

Pyridoxal-5′-phosphate (PLP), the active coenzyme form of vitamin B6, is essential for a wide range of metabolic and neurological functions. It plays a key role in amino acid metabolism—particularly in the synthesis of gamma-aminobutyric acid—as well as in folate and one-carbon metabolism, protein and polyamine biosynthesis, carbohydrate and lipid metabolism, mitochondrial function, and erythropoiesis. 1

A growing number of rare inborn errors of metabolism have been identified in which impaired PLP availability leads to epileptic encephalopathy. These include pyridox(am)ine-5′-phosphate oxidase (PNPO) deficiency, ALDH7A1 deficiency, PLP-binding protein deficiency, hyperprolinemia type II, hypophosphatasia, and defects in glycosylphosphatidylinositol anchor biosynthesis. 2 3

Despite their distinct genetic backgrounds, these disorders typically present with early-onset seizures that respond to either pyridoxine or PLP treatment. Most patients develop symptoms during the neonatal or early infantile period, although a minority may present later. The severity of the seizures and associated neurodevelopmental outcomes varies considerably, from mild to severe. Other shared clinical features may include developmental delay and systemic findings such as anemia, lactic acidosis, or hypoglycemia. 2

Empirical treatment with pyridoxine (30 mg/kg/d orally) and/or PLP (30 mg/kg/d orally) is commonly used in the diagnostic workup of suspected B6-dependent epilepsies. 1 4 A positive response to therapy is considered both diagnostically and therapeutically valuable, although the clinical improvement may be delayed, even in patients later confirmed to have a genetic diagnosis. Long-term supplementation with pyridoxine or PLP often leads to effective seizure control, though its impact on cognitive and systemic symptoms tends to be more modest. 5 6

In PNPO deficiency the majority of patients only respond to PLP and are therefore dependent on this medication. 7 There is growing evidence that PLP is a risk factor for the development of liver cirrhosis and hepatocellular carcinoma (HCC). 8 9 10 Although there were also hypotheses suggesting that it is not only the toxicity of PLP, but that the disease itself may also cause liver problems such as in tyrosinemia type 1. 9 In some cases—while awaiting further genetic or biochemical diagnostic work-up—it is difficult to distinguish between the different forms of vitamin B6-dependent epilepsy, leading clinicians to initiate a trial of PLP in patients who may have an alternative B6-responsive disorder such as ALDH7A1 deficiency. 11 Such case-based observations may help to disentangle the potential hepatotoxic effects of PLP from the contribution of the underlying disease itself in the development of liver pathology. Here, we present four patients with B6-dependent epilepsy who were treated with oral PLP and subsequently developed liver disease. We explore the possible pathomechanisms underlying this association, including both PLP-induced hepatotoxicity and potential intrinsic hepatic involvement related to the metabolic defect itself.

### Patient 1

This now 9-year-old boy with genetically confirmed PNPO deficiency has been on PLP monotherapy since the age of 17 months for seizure control. Prior to that, he was treated with pyridoxine. His initial presentation, previously reported, 7 12 involved neonatal-onset seizures that were unresponsive to standard antiseizure medication (ASM) but ceased 24 hours after initiating intravenous pyridoxine (15 mg/kg/d). Subsequent withdrawal of ASM was successful.

Biochemical findings during pyridoxine treatment suggested PNPO deficiency, with elevated cerebrospinal fluid (CSF) pyridoxal and borderline PLP levels. Genetic testing revealed a homozygous missense pathogenic variant in the PNPO gene (c.481C > T; p.Arg161Cys); both parents were heterozygous carriers.

Despite the genetic diagnosis, pyridoxine monotherapy was initially continued due to the observed clinical response and concerns over PLP-related hepatotoxicity and possible paradoxical reactions. At 17 months, treatment was switched to PLP (30–40 mg/kg/d) following increased seizure activity and two episodes of status epilepticus. PLP monotherapy resulted in effective seizure control as long as it was given strictly on time. The brand of PLP was frequently changed due to supply issues with various providers. He had a developmental disability and attended special education.

**Fig. 1:**
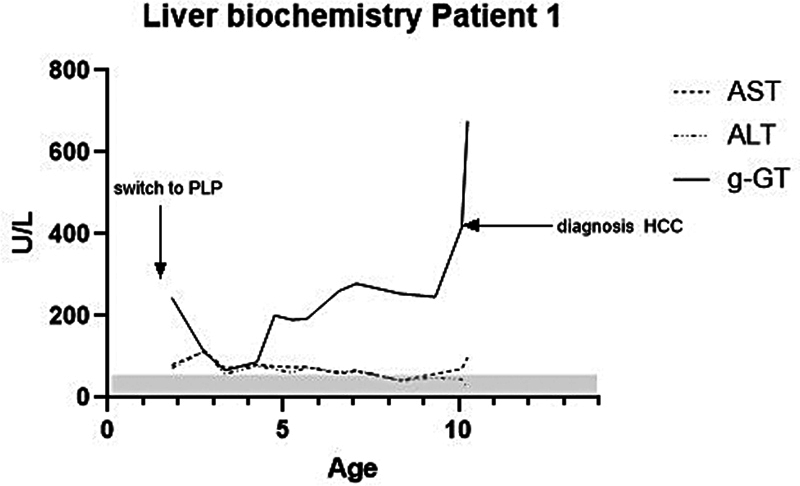
Longitudinal liver enzyme levels in Patient 1 with PNPO deficiency. Plasma levels of AST (aspartate aminotransferase), ALT (alanine aminotransferase), and γ-GT (gamma-glutamyl transferase) are plotted over time, with age in years on the *x* -axis and enzyme levels in U/L on the *y* -axis. The initiation of pyridoxal 5′-phosphate (PLP) treatment is indicated with an arrow labeled “switch to PLP.” A second arrow denotes the time of hepatocellular carcinoma (HCC) diagnosis. The shaded area represents the upper limit of the normal reference range for γ-GT. Elevated and rising γ-GT levels are observed following the initiation of PLP therapy, preceding the development of cirrhosis and subsequent HCC.

Liver function tests were regularly monitored and showed stable aspartate aminotransferase (AST) and alanine aminotransferase (ALT) values, whereas gamma-glutamyl transferase (GT) levels remained persistently elevated (ranging from 65 to 250 U/L; Fig. 1 ). At the age of 6, liver ultrasound findings were unremarkable with no evidence of cirrhosis. However, follow-up imaging 2 years later revealed the development of cirrhosis accompanied by splenomegaly. Two years after this diagnosis, the patient presented with weight loss, difficulty swallowing, and right leg pain. Laboratory analysis revealed markedly elevated alpha-fetoprotein (aFP) levels (500,000 µg/L, normal <7 µg/L), and imaging confirmed the presence of HCC with metastatic spread to the sacral spine. It is noteworthy that an ultrasound performed 6 months earlier had still shown stable cirrhotic changes without signs of malignancy. The patient is currently receiving palliative care, as no further treatment options are available due to metastatic disease. Since the HCC diagnosis, efforts have been made to gradually reduce the total daily PLP dose by increasing dosing frequency. The current dosage is 31 mg/kg/d, reflecting a 21% reduction, with further tapering still being attempted.

### Patient 2

This 8-year-old boy was diagnosed with PNPO deficiency in the neonatal period after presenting with treatment-resistant seizures within hours after birth. He is the first child of nonconsanguineous parents. Initial ASM therapy was unsuccessful and a trial with intravenous pyridoxine (initially 50 mg/kg/d, later reduced to 22 mg/kg/d) proved ineffective. On day 3 of life, oral PLP was initiated (30 mg/kg/d via nasogastric tube), resulting in prompt cessation of seizures. 7

Biochemical testing supported the diagnosis of PNPO deficiency, with low CSF pyridoxal levels (<20 nmol/L; reference: 20–93), undetectable PLP (reference: 30–49 nmol/L), and normal urinary alpha-aminoadipic semialdehyde (AASA; 469 nmol/mmol creatinine; reference: 270–2,850). Genetic testing confirmed compound heterozygous pathogenic variants in PNPO: c.364-1G > A (a splice-site variant) and c.448_451del [p.Pro150Argfs*27], both previously reported in association with PNPO deficiency.

Following discharge, the patient remained on PLP monotherapy at a dose of 40 mg/kg/d. Over the years, several changes in PLP formulation were necessary due to availability issues and regulatory restrictions, including a 2018 Dutch limit on vitamin B6 content in supplements. Some formulations led to adverse effects such as abnormal behavior or breakthrough seizures, prompting a return to the previously tolerated preparation.

Liver enzymes have been monitored regularly.

At age 6, abdominal ultrasound revealed cirrhosis and splenomegaly. Eighteen months later, a magnetic resonance imaging (MRI) prompted by elevated aFP (7,000 µg/L; normal <7 µg/L) identified multiple hepatic lesions suspicious for HCC, whereas a previous MRI 8 months earlier had shown no signs of HCC. The patient underwent local ablation of the lesions, and no metastatic disease was detected. Following the HCC diagnosis, a successful attempt was made to taper the PLP dosage by increasing the dosing frequency, allowing a reduction in the total daily dose. The patient is currently maintained on 28 mg/kg/d, representing a 30% reduction.

### Patient 3

This 5-year-old boy is the second child of a Moroccan consanguineous couple. Pregnancy was marked by oligohydramnios and spontaneous premature birth at AD 36 + 1. He presented within the first hour of life with seizures and hyperthermia. Antibiotic therapy, ASM, and vitamins were initiated, with phenobarbital, pyridoxine (dosage not available), biotin, and folate. At 3 days, seizures were still ongoing, and phenobarbital was replaced by levetiracetam (50 mg/kg/d).

The first biochemical testing showed elevated vanillactic acid in urine and elevated B6-sensitive neurotransmitters in the CSF, both indicating either a PNPO or antiquitin deficiency. Urinary AASA was not preformed.

PLP (50 mg/kg/d) was initiated at 9 days of life and resulted in seizure control. By 1 month, he still received levetiracetam (50 mg/kg/d) and PLP (50 mg/kg/d), and other vitamins were stopped, with no seizure recurrence.

PNPO deficiency was suspected because seizures were unresponsive to pyridoxine but ceased with PLP (plus levetiracetam). At age 5 months, a systematic check-up showed slightly elevated liver enzymes, with AST at 81 IU/L (normal < 74 IU/L) and ALT at 65 IU/L (normal < 42 IU/L). At that time he was still treated with PLP (42 mg/kg/d) and levetiracetam (40 mg/kg/d). At age 9 months, a mild neurodevelopmental delay was observed (sitting unsupported had not been acquired, and he showed difficulties with the thumb–index finger grip) with no other clinical abnormalities, especially no hepatomegaly. Seizures were controlled by the same two medications, with a slight diminution in PLP dosage (36 mg/kg/d). In this setting, laboratory parameters showed marked elevation in liver enzymes (AST: 315 UI/L ; ALT: 199 UI/L), cholestasis (alkaline phosphatase: 393 IU/L [normal < 320] and gamma-GT 83 IU/L [normal < 25]) with normal bilirubin. Alpha-FP was elevated at 4,000 μg/L (normal < 10). Liver function (clotting factors) was normal. Abdominal ultrasound showed hepatomegaly with an increased liver span at 10 cm. At age 10 months, the patient started to show hypoketotic hypoglycemia after short fasting, no triggering event was noticed. Hepatomegaly was clinically noticed and appeared to be rapidly progressive. Additionally, because of concern for hyperammonemia, PLP and levetiracetam were temporarily withheld and restarted 24 hours later after normal ammonia levels were confirmed, with no seizures recurrence. A liver inborn error was therefore suspected.

Results of whole exome sequencing (WES) became available and showed homozygous mutation in *ALDH7A1* (c.1279G > C ; p.Glu427Gln), with both parents being heterozygous. No variants in PNPO nor in liver metabolic diseases genes were identified.

Treatment was switched from PLP to pyridoxine 36 mg/kg/d over a 2-day period, and fasting time was limited with the introduction of nocturnal enteral nutrition.

At age 12 months, liver tests started to normalize, hepatomegaly normalized, and no new episode of hypoglycemia was registered, which allowed withdrawal of nocturnal enteral nutrition at age 13 months. At age 16 months, liver parameters were fully normal without any recurrence of hypoglycemia.

Compliance to oral pyridoxine was perfect, and levetiracetam could be discontinued at age 18 months, with no seizure recurrence. Liver size and liver parameters remained persistently normal (aFP: 15 μg/L, normal < 35) along with liver ultrasound.

Even if such a reversible liver disease was most likely due to PLP hepatotoxicity, a whole genome sequencing was performed to rule out putative intronic variants that would have been missed by WES.

At last follow-up at age 4 years and 7 months, liver function and all liver parameters are normal. Regarding the neurodevelopmental delay, the patient continues to show progress, but still has a mild delay for his age.

### Patient 4

This boy with ALDH7A1 deficiency presented at 8 months of age with ASM-refractory status epilepticus requiring prolonged management in the intensive care unit (ICU). He received intravenous pyridoxine with no clinical response and was maintained on daily pyridoxine for several months along with multiple ASMs. Pyridoxine was eventually discontinued but then restarted after an analysis of CSF neurotransmitters noted the presence of “peak X” characteristic of ALDH7A1 deficiency. 13 ALDH7A1 deficiency was eventually confirmed demonstrating homozygous c.1279G > C; p.Glu427Gln variants in *ALDH7A1* . Despite reinstitution of pyridoxine, he continued to have poor control of seizures, and he required the use of multiple ASMs. Folinic acid was added to his regimen as was PLP, but seizure control remained incomplete. He had significant neurodevelopmental impairment and required the use of a feeding tube.

Between 2 and 3 years of age his folinic acid, pyridoxine, and PLP were sequentially discontinued with no overall change in his seizure frequency. However, he was then diagnosed with hydrocephalus a ventriculoperitoneal shunt was placed. During his ICU hospitalization for management of hydrocephalus, he subsequently experienced significant agitation and respiratory distress (though no increased seizures on electroencephalogram [EEG] monitoring) and intravenous pyridoxine was administered. This led to apnea and a completely suppressed EEG background lasting several days before slowly recovering. Months later, over 24 to 48 hours, he suddenly transitioned from being diffusely hypotonic to profound hypertonia. After failing oral antispasticity medications, he required placement of an intrathecal baclofen pump. He was subsequently treated with levetiracetam, clobazam, and folinic acid together with pyridoxine and PLP. Each dose of PLP was crushed with a mortar and pestle and mixed into a slurry with a small amount of liquid to be administered via the feeding tube. Despite these ASMs and vitamin B6 preparations, he continued to have multiple short tonic seizures daily. Folinic acid was eventually discontinued, and he was maintained on pyridoxine 63 mg/kg/d and PLP 25 mg/kg/d. He remained on the same brand of PLP over the entire course of treatment.

At 7 years of age elevated liver transaminase levels were noted. At that time, imaging studies and a liver biopsy were unremarkable. He subsequently developed ascites and was then diagnosed with HCC. The tumor was inoperable, and he was treated with chemotherapy. His course was complicated by worsening ascites, and he expired at 9 years of age.

## Discussion

The development of HCC and rapidly evolving hepatotoxicity in three of the four patients described in this series raises significant concerns regarding the long-term safety of PLP supplementation in patients with inborn errors of vitamin B6 metabolism. Although PLP is widely considered a safe and essential cofactor, its chronic high-dose use in rare metabolic conditions such as PNPO or ALDH7A1 deficiency may pose hepatotoxic risks. 9

Importantly, for most patients with PNPO deficiency, PLP is the only effective treatment, as pyridoxine cannot be converted to its active form due to the enzymatic defect. 6 These patients are therefore fully dependent on lifelong PLP supplementation to maintain seizure control. This therapeutic necessity leaves little flexibility for dose reduction, making the risk of hepatotoxicity particularly concerning. In two of our patients (Cases 3 and 4), diagnostic delay occurred because the absence of clinical response to pyridoxine initially misled clinicians—an issue that has been reported previously in vitamin B6–dependent epilepsies. 14

Initially, PNPO deficiency itself was hypothesized to be a potential risk factor for liver disease and consequent HCC. 9 As was pointed out in a recent review, 30% of published patients with PNPO deficiency show liver involvement. 15 However, two of our patients had ALDH7A1 deficiency—who also received high-dose PLP—developed either severe hepatatoxicity or HCC, pointing instead to PLP exposure as the likely common denominator. This finding is supported by a case report of a homocystinuria patient with induced liver injury caused by PLP. 16 As is shown in Table 1 , the exposure to PLP was at least 5 years before the development of HCC.

**Table 1:** Overview of patients and pyridoxal 5′-phosphate exposure

Patient number	Highest dose PLP mg/kg/d	Highest level γ-GT (U/L)	Years of exposure until occurrence HCC	Current (lowest) dose (mg/kg/d; freq/d)
1	40	664	8.8	31–5/d
2	41	100	7.6	28–5/d
3	50	83	NA	36–3/d
4	50	135	∼5	25–2/d

Abbreviations: γ-GT, gamma-glutamyl transferase; HCC, hepatocellular carcinoma; NA, not applicable; PLP, pyridoxal-5′-phosphate.

The exact mechanism behind the observed hepatotoxicity associated with PLP supplementation remains poorly understood. One proposed explanation is that PLP or its breakdown products may exert toxic effects, particularly when supplement quality is inconsistent—as is often the case with over-the-counter formulations used in PNPO deficiency. 7 Coman et al. hypothesized that high doses of PLP could provoke fibrogenic activation of hepatic stellate cells, possibly through purinoceptor-mediated signaling pathways, thereby promoting progression toward cirrhosis. 9

The chemical reactivity of PLP is also a key factor. As an active aldehyde, PLP is capable of forming Schiff bases with amino groups in proteins and other metabolites, leading to spontaneous chemical modifications such as epimerization and lysine adduct formation, both of which may alter protein function and stability. 17 A comparable mechanism has been demonstrated with acetaldehyde, another reactive aldehyde, which interferes with hepatic enzyme activity—specifically Δ4-3-oxosteroid 5β-reductase—by modifying lysine residues, resulting in toxic metabolite accumulation and liver injury. 18 These parallels suggest that PLP could act through similar pathways to impair hepatic enzymatic systems.

Another possible contributor to toxicity is microbial degradation of excess PLP in the gut. Although many gut bacteria synthesize vitamin B6 vitamers, they may also degrade surplus PLP into α,β-unsaturated carbonyl intermediates, compounds known for their cytotoxicity. Inherited disorders such as tyrosinemia type 1 involve similar compounds (e.g., fumarylacetoacetate), which are directly implicated in liver failure and hepatocarcinogenesis. 19 20 Additionally, a PLP photodegradation product, 4-pyridoxic acid 5′-phosphate, has been suggested as a potential inhibitor of PLP-dependent enzymes. 21 This compound is normally absent in human metabolism but may form under improper storage or during manipulation of PLP preparations. Compounding these biochemical risks is the lack of standardization in PLP formulations. Due to regulatory and supply constraints, patients frequently switch between brands of uncertain purity, with variable excipients and bioavailability as shown in our first two described patients. Clinical observations in our cohort—including breakthrough seizures or behavioral changes after formulation changes—highlight this issue. It is important to note that in the first two patients described, the total daily PLP dose could be reduced by more than 30% simply by increasing the dosing frequency from three to five times per day. This adjustment represents a significant step toward minimizing potential hepatotoxicity while maintaining seizure control.

Given the essential role of PLP in these patients, there is a critical need to develop alternative delivery routes that may bypass hepatic first-pass or gut metabolism, such as intranasal administration. Chronic intravenous use is impractical for lifelong therapy. Until safer formulations or routes are established, routine hepatic monitoring and strict quality control of PLP preparations are vital.

Given the emerging evidence for PLP-associated hepatotoxicity, chronic PLP therapy should only be initiated in patients with a confirmed genetic diagnosis for whom no alternative treatment options exist. For all patients receiving long-term PLP, structured hepatic surveillance every 3 to 6 months is warranted, including at minimum monitoring of AST, ALT, gamma-GT, coagulation parameters, α-fetoprotein, and liver ultrasound. 22
